# Quadriceps and Hamstring Muscle Strength Recovery After a Multiligament Knee Injury: A Long-term Follow-up

**DOI:** 10.1177/03635465261466381

**Published:** 2026-08-12

**Authors:** Ingrid Trøan, Inger Holm, Gilbert Moatshe, Robert F. LaPrade, Lars Engebretsen, Tone Bere

**Affiliations:** * Orthopaedic Division, Oslo University Hospital, Oslo, Norway; † Oslo Sports Trauma Research Center, Norwegian School of Sports Sciences, Oslo, Norway; ‡ University of Oslo, Oslo, Norway; ‖ Twin Cities Orthopedics, Edina, Minnesota, USA; Investigation performed at Orthopaedic Division, Oslo University Hospital, Oslo, Norway

**Keywords:** multiligament knee injury, quadriceps muscle strength, hamstring muscle strength, physical function

## Abstract

**Background:**

Multiligament knee injuries (MLKIs) are serious and heterogeneous injuries that often lead to long-term functional limitations. There is limited literature regarding objective muscle strength and functional recovery after surgical treatment of MLKIs.

**Purpose:**

To investigate long-term quadriceps and hamstring muscle strength and functional recovery after surgical treatment of MLKI and to compare postoperative outcomes between single cruciate MLKI and bicruciate MLKIs.

**Study Design:**

Cross-sectional study; Level of evidence, 3.

**Methods:**

Patients surgically treated for an MLKI at a single level 1 trauma center between January 2013 and December 2020 were included. Isokinetic quadriceps and hamstring muscle strength were assessed using a Biodex dynamometer, and physical function was measured by single-leg hop, triple hop, crossover hop, and 6-m timed hop tests, with the limb symmetry index (LSI) calculated.

**Results:**

Of 124 eligible patients, 90 (72%) participated at a mean follow-up time of 7 years (SD, 2.1) since surgery to evaluation. The median age at the time of testing was 49 years (range, 21-72) and 59% were male. In total, 54% of the patients had not regained full quadriceps strength (LSI <90%), and 46% had persistent hamstring deficits for peak torque/body weight (PT/BW) at 60 deg/s. Across the entire cohort, there was a significant difference between the uninvolved and involved sides for quadriceps and hamstrings for PT/BW (*P* = .01). The mean LSI for quadriceps PT/BW was 88% at 60 deg/s and 90% at 240 deg/s, while for the hamstrings, the mean LSI was 93% at 60 deg/s and 97% at 240 deg/s. Median LSIs for the hop tests ranged from 93% (range, 41%-187%) for single leg to 99% (42%-141%) for crossover; however, 44% of patients failed to reach the 90% threshold for the single-leg hop. The 6-m timed hop test and quadriceps muscle strength were significantly lower for patients with bicruciate injuries than those with single cruciate injuries (*P* < .05).

**Conclusion:**

Persistent deficits in quadriceps and hamstring muscle strength were observed 7 years after MLKI surgery, with a substantial proportion of patients failing to regain full functional symmetry in muscle strength and hop test performance. Patients with bicruciate injuries demonstrated significantly more pronounced deficits in muscle strength and hop test scores as compared with those with single cruciate injuries.

Multiligament knee injuries (MLKIs) represent some of the most devastating knee injuries and often affect patients’ ability to return to their previous levels of function in sports participation and at work. An MLKI usually affects the young and active population, involving the disruption of ≥2 major knee ligaments,^23,27,39^ potentially leading to instability, pain, and long-term functional limitations. Owing to the severity of MLKIs, these injuries often present with concomitant injury to surrounding structures, including neurovascular injuries, which can significantly affect patient outcomes. The quadriceps and hamstring muscles play a critical role in knee stability and lower limb function, and adequate strength in these muscle groups is essential for daily activities and mobility after knee surgery. Weakness in these muscle groups can significantly impair activities such as walking, stair climbing, rising from a chair, and returning to sport or work.^16,29^ Residual quadriceps weakness is associated with impaired knee stability, reduced function, and long-term disability. It may also contribute to excessive mechanical stress on articular cartilage, potentially accelerating degenerative changes and osteoarthritis progression.^1,30^ These challenges are particularly pronounced after severe knee trauma such as an MLKI.^9^

Isokinetic muscle strength testing and hop performance^2^ have been widely used for decades to assess quadriceps and hamstring strength in athletic and nonathletic populations.^6,21,38^ However, such assessments are infrequently reported in the MLKI population. The limited studies available^7,10,11,16^ have primarily focused on short-term follow-up, finding that strength and function may improve within the first 1 to 2 years postoperatively. These studies note that strength deficits frequently persist beyond 24 months, although data on whether these strength deficits are sustained over the long term are still lacking. Understanding muscle strength deficits in patients with MLKI will provide valuable insights into optimizing rehabilitation strategies, potentially improving long-term functional outcomes, and delaying degenerative changes. Furthermore, while information on functional recovery is reported for single cruciate MLKIs and bicruciate MLKIs,^7,11^ it rarely provides direct comparisons between these groups, owing to small sample sizes and heterogeneous injury patterns.^10^ Given the limited documentation on muscle strength and functional recovery in this population, the purpose of this study was to investigate intermediate-term quadriceps and hamstring muscle strength and functional recovery after surgical treatment of MLKI and to compare postoperative outcomes between single cruciate and bicruciate MLKIs.

## Methods

### Patient Population

This cross-sectional study consisted of patients who underwent surgical management for MLKI at the orthopaedic department of a level 1 trauma center between January 2013 and December 2020. The study was approved by the Regional Committee for Medical and Health Research Ethics (REK Nord-IRB206446). Informed consent was received from all included patients. MLKIs were defined as injuries to at least 2 of the 4 major knee ligaments requiring surgical intervention: the anterior cruciate ligament (ACL), posterior cruciate ligament (PCL), medial collateral ligament (MCL), or fibular collateral ligament (FCL). Eligible patients had at least 24 months of postoperative follow-up. Exclusion criteria included patients who had undergone revision ligament reconstruction, tibial osteotomy, or total knee replacement before the time of follow-up, as well as those with concomitant intra-articular knee fractures requiring surgical treatment. Of the 191 patients treated during the study period, 124 (65%) agreed to participate in an initial study assessing self-reported function.^36^ From this group, 90 patients consented to participate in the present study, evaluating muscle strength and physical function.

### Surgical Treatment

All patients underwent anatomic reconstructions based on established guidelines,^3,26,36^ performed at a single center by surgeons experienced in treating MLKIs. The ACL reconstructions were performed with either a bone–patellar tendon–bone or hamstring tendon autograft. For PCL tears, a double-bundle anatomic PCL reconstruction was performed with Achilles, tibialis anterior, or tibialis posterior tendon allografts. The MCL was reconstructed via the LaPrade repair augmentation technique with autograft,^1^ and the LaPrade posterolateral corner reconstruction technique was used for posterolateral corner injuries with allograft.^22^

### Postoperative and Rehabilitation Protocol

After surgery, patients with PCL involvement were immobilized in a knee range of motion (ROM) brace locked in full extension. Passive extension was initiated immediately, and after swelling subsided, the brace was replaced with a dynamic PCL brace. This brace was worn for 12 to 16 weeks, with initial settings that limited the knee ROM to 0° to 90°. For patients without PCL injuries, a ROM brace was used for 8 weeks, allowing full ROM. All patients followed a rehabilitation program that consisted of initial partial weightbearing with crutches, early isometric quadriceps training, and straight-leg exercises. After 8 weeks, full weightbearing and progressive strengthening activities, such as stationary cycling, were introduced. From 16 weeks postoperatively, rehabilitation focused on restoring full motion, improving muscular control, and advancing functional training, such as walking, stair work, and balance exercises. Neuromuscular training was gradually progressed from stable to dynamic conditions, and strength training was intensified with high-load and endurance-based protocols. Light jogging was introduced once patients demonstrated full mobility, adequate quadriceps control with a ≥75% quads limb symmetry index (LSI), and the ability to walk 30 minutes without pain or increased swelling. Return to full activity was permitted and recommended when patients achieved at least 90% quadriceps strength symmetry as compared with the contralateral limb and nearly full knee motion.

### Baseline Characteristics

A retrospective chart review was conducted to obtain baseline variables: age at the time of injury, sex, date of injury, side of injury, type of injury, presence of meniscal and cartilage injuries, surgical procedures, treatment, and complications. The ligament injuries were categorized according to the modified Schenck classification, as outlined by Poploski et al,^31^ which differentiates various MLKI patterns (MLK 1-AM to MLK 4) depending on the ligaments involved. For the analysis, cases were grouped into 5 types: type 1 (single cruciate with MCL or FCL), type 2 (bicruciate), type 3-L (ACL, PCL, FCL), type 3-M (ACL, PCL, MCL), and type 4 (all major ligaments involved). Injuries were further stratified by energy mechanism: high-energy events included motor vehicle accidents, significant falls (>5 ft), or downhill skiing, whereas low-energy injuries occurred during ball sports, cross-country skiing, minor falls (<5 ft), or daily activities.

### Muscle Strength Testing and Physical Function

A Biodex System 4 dynamometer with Advantage BX software (Version 5.3.06) was used to assess isokinetic quadriceps and hamstring muscle strength. Before the test, participants performed a 5-minute warm-up on an ergometer cycle. After the warm-up session, the participant was secured to the apparatus with straps across the chest, pelvis, thigh, and ankle joint. Before data collection, the dynamometer's gravity correction procedure was performed according to the manufacturer's recommendations to account for the weight of the tested limb. Participants were then given 5 to 10 trial repetitions to familiarize themselves with the system. The test protocol consisted of 5 maximal repetitions at 60 deg/s to assess peak torque capacity, followed by a 1-minute rest period and 30 repetitions at 240 deg/s to evaluate muscular endurance and fatigue resistance, in accordance with the Biodex manufacturer guidelines. The uninvolved side was tested first, and the ROM was set from 90° of flexion to 0° of extension, depending on the patient's ROM. Body weight–corrected peak torque (PT/BW) and total work (TW) values were used in the analysis. Of the 90 patients included in the study, 88 completed the strength tests. To measure physical function, the participants performed 4 hop tests: single hop for distance, triple hop for distance, triple crossover hop, and 6-m timed hop. These tests serve as proxies for neuromuscular control, strength, and confidence in the limb^25,28,32,35^ and are commonly used to assess ligament injuries.^28^ Of the 90 included patients, 15 to 17 were unable to complete the hop tests because of difficulties performing them. The 2 patients who did not complete the strength testing were nevertheless able to perform the hop tests.

### Statistical Analysis

Statistical analyses were performed in Stata SE 18 (Stata Corp LLC). Patient characteristics and surgical details were presented as mean with standard deviation or median with range for continuous variables and as frequency and percentage for categorical variables. The groups were compared by *t* test for continuous variables and Wilcoxon rank sum (Mann-Whitney) test for continuous, nonnormally distributed variables. A *P* value of .05 was considered statistically significant, and all tests were 2-sided. LSI was calculated by dividing the result for the involved leg by that of the uninvolved leg and multiplying by 100, based on PT/BW and TW values. Normalizing the strength to body weight allows for more clinically meaningful comparisons among individuals rather than absolute torque values alone and is consistent with the primary strength outcomes of this study. An LSI ≥90% was considered normal.^5,28^ To evaluate the balance between muscle groups, the hamstrings-quadriceps (H/Q) ratio representing the relationship between hamstring and quadriceps strength was calculated. The ratio is calculated by dividing peak torque of the hamstring muscle by peak torque of the quadriceps muscle.^33^ Additional post hoc correlation analyses were performed using patient-reported outcome measures and return-to-sport data from the same study cohort, previously reported elsewhere,^36,37^ to examine associations with muscle strength measurements using Spearman ρ (*P* < .05).

## Results

### Patient Demographics

Of the 90 patients, 59% were male with a mean (SD) body mass index of 28 (8.1) at the time of testing. The median age was 49 years (range, 21-72), and the mean time from surgery to evaluation was 7 years (2.1). Most participants (n = 65, 72%) had sustained low-energy injuries versus high-energy injuries (n = 25; 28%). The injury mechanisms and injury patterns according to the modified Schenck classification are reported in Table 1. Concomitant chondral injuries were present in 32 knees (35%) and meniscal tears in 27 (30%). Peroneal nerve injury occurred in 6 patients (7%), with 5 (5%) undergoing a tibialis posterior tendon transfer for a foot drop.

**Table 1:** Injury Mechanism and Injury Pattern*^*a*^*

	No. (%)
Injury mechanism	
Motor vehicle accident	1 (1)
Motorcycle	7 (8)
Ball sports	14 (16)
Skiing sports	30 (33)
Other sports	14 (16)
Other*^*b*^*	24 (26)
Multiligament injury pattern	
MLK 1-AM: ACL and MCL	19 (21)
MLK 1-AL: ACL and FCL	18 (20)
MLK 1-AML: ACL, MCL, FCL	0 (0)
MLK 1-PM: PCL and MCL	3 (3)
MLK 1-PL: PCL and FCL	4 (3)
MLK 1-PML: PCL, MCL, FCL	1 (1)
MLK 2: ACL and PCL	8 (9)
MLK 3-M: ACL, PCL, MCL	26 (29)
MLK 3-L: ACL, PCL, FCL	9 (10)
MLK 4: ACL, PCL, MCL, FCL	2 (1)

a ACL, anterior cruciate ligament; FCL, fibular collateral ligament; MCL, medial collateral ligament; MLK, multiligament knee; PCL, posterior cruciate ligament.

b Injury mechanisms classified as “other” include falling on a slippery floor, falling from a height, getting run over by a dog, and tripping.

### Quadriceps and Hamstring Muscle Strength

**Figure 1.**
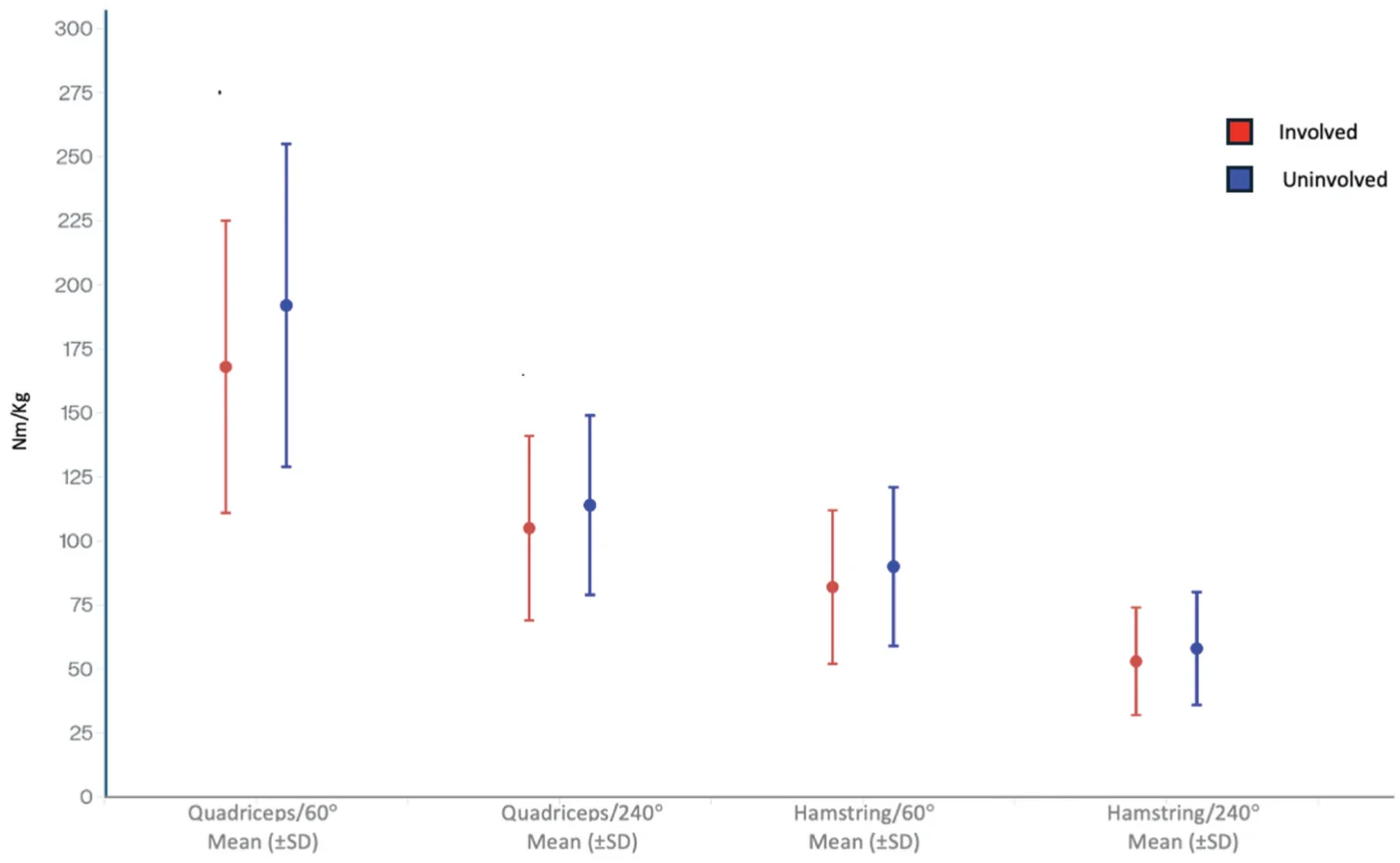
Body weight–corrected peak torque for quadriceps and hamstring at 2 angular velocities comparing involved and uninvolved limbs. Error bars represent the standard deviation.

For the quadriceps PT/BW at 60 deg/s, the mean (SD) was 168 (57) N·m/kg for the involved limb versus 192 (63) N·m/kg for the uninvolved limb (side-to-side difference, 24 ± 39; *P* = .01), while at 240 deg/s, it was 105 (36) versus 114 (35) N·m/kg, respectively (side-to-side difference, 9 ± 18; *P* = .01). For hamstring PT/BW at 60 deg/s, the mean (SD) was 82 (30) N·m/kg for the involved limb versus 90 (31) N·m/kg for the uninvolved limb (side-to-side difference, 8 ± 16; *P* = .01), while at 240 deg/s, it was 53 (21) versus 58 (22) N·m/kg (side-to-side difference, 5 ± 12; *P* = .01) (Figure 1). Results for the TW capacity are presented in Table 2.

**Table 2:** Total Work in Joules for Quadriceps and Hamstring Muscles at the 2 Angular Velocities (60 and 240 deg/s)

	Limb, J, Mean ± SD			
Total Work: Muscle	Involved	Uninvolved	Difference	*P* Value
60 deg/s				
Quadriceps	659 ± 241	743 ± 279	83 ± 154	<.001
Hamstrings	357 ± 165	398 ± 176	40 ± 84	<.001
240 deg/s				
Quadriceps	1816 ± 758	1969 ± 748	144 ± 338	<.001
Hamstrings	808 ± 411	848 ± 403	40 ± 215	.03

**Figure 2.**
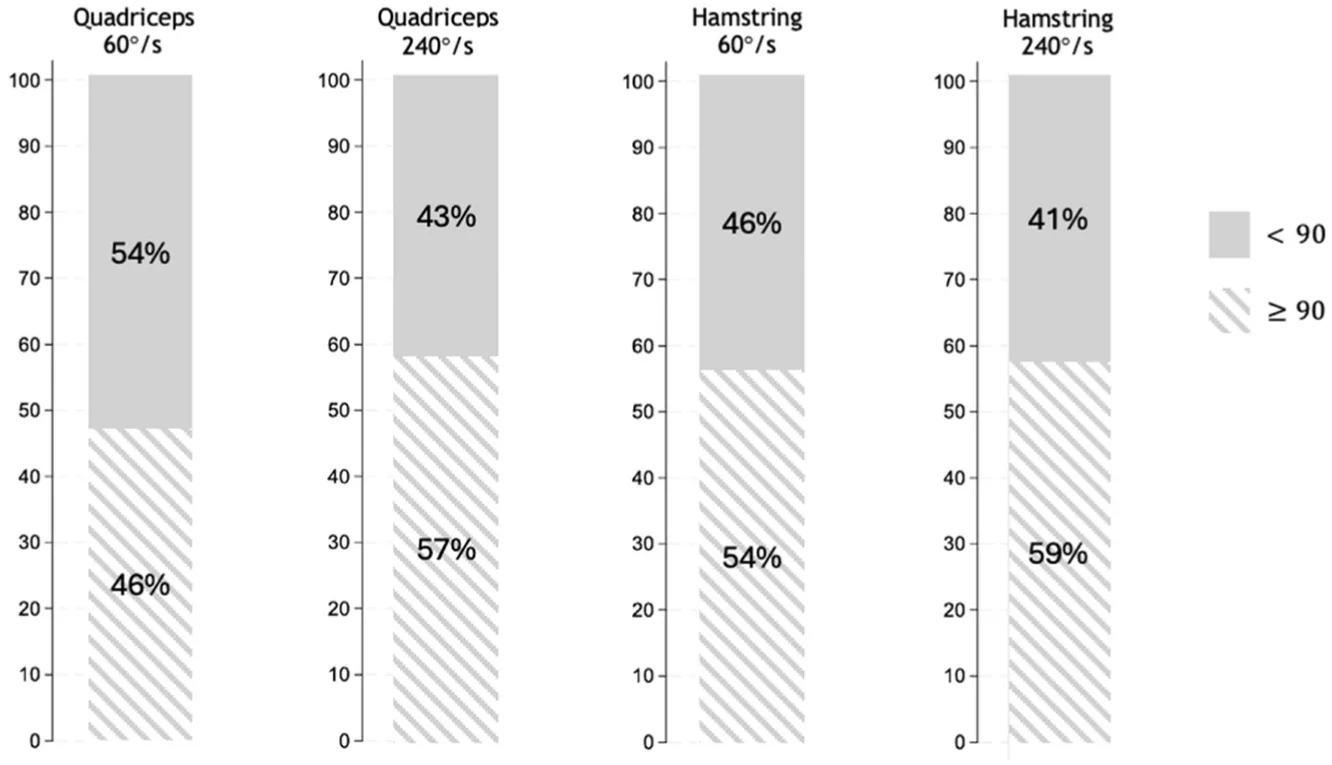
Percentage of participants with normal (LSI ≥90%) and abnormal (LSI <90%) limb symmetry for peak torque body weight for quadriceps and hamstrings at 60 and 240 deg/s (n = 88). LSI, limb symmetry index.

The mean (SD) quadriceps PT/BW LSI was 87% (19%) at 60 deg/s and 92% (16%) at 240 deg/s, while the mean hamstring LSI was 91% (24%) and 91% (21%), respectively. Figure 2 presents the percentage of patients with normal limb symmetry (LSI ≥90%) for PT/BW for quadriceps and hamstrings at 60 and 240 deg/s. For the TW, the corresponding proportions were 42% (n = 37) for the quadriceps LSI at 60 deg/s, 56% (n = 50) for the quadriceps LSI at 240 deg/s, 52% (n = 46) for the hamstring LSI at 60 deg/s, and 59% (n = 52) for the hamstring LSI at 240 deg/s. Table 3 presents the mean H/Q ratio for PT/BW and TW.

**Table 3:** Mean Hamstring to Quadriceps Ratio

	Limb, Mean ± SD	
Ratio	Involved	Uninvolved
Peak torque/body weight		
60 deg/s	0.49 ± 0.11	0.51 ± 0.12
240 deg/s	0.51 ± 0.12	0.53 ± 0.13
Total work		
60 deg/s	0.50 ± 0.14	0.53 ± 0.15
240 deg/s	0.44 ± 0.15	0.43 ± 0.15

### Hop Tests

**Figure 3.**
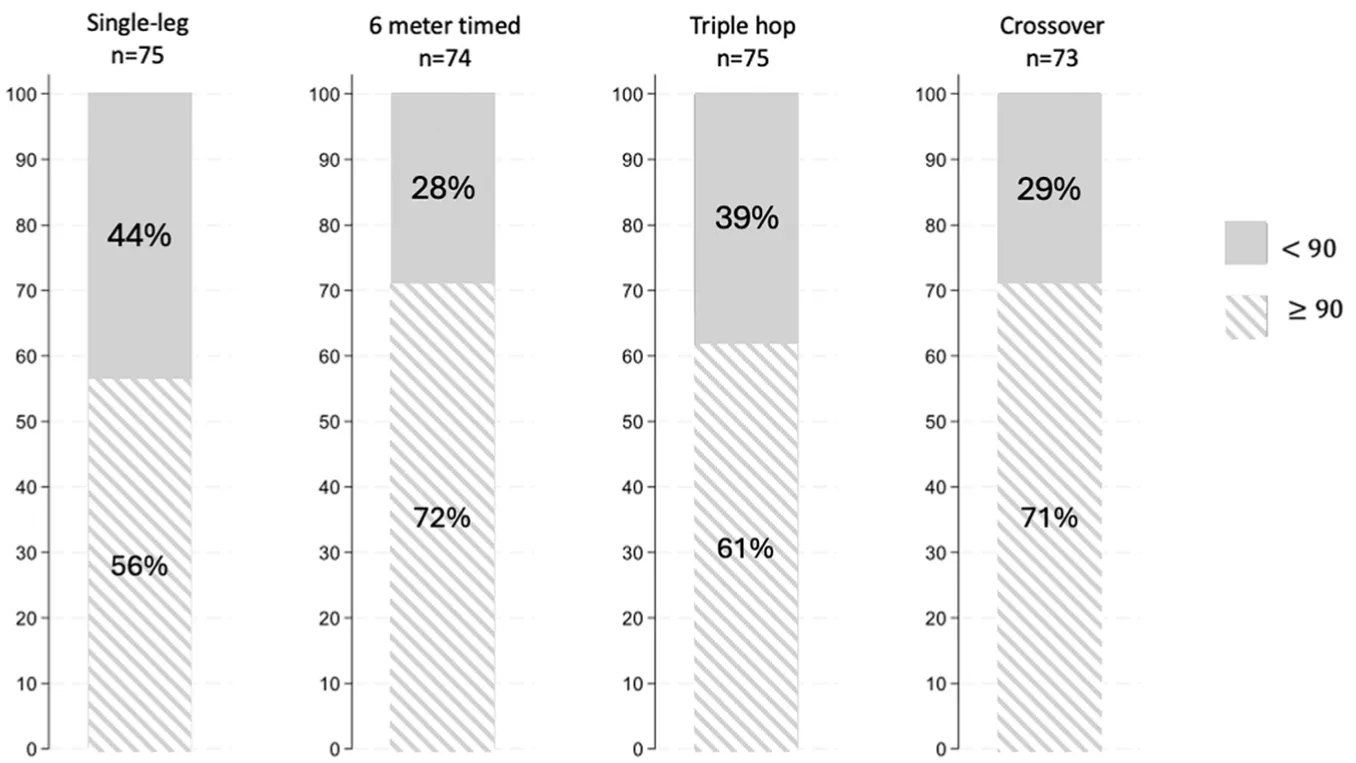
Percentage of participants with normal (LSI ≥90%) and abnormal (LSI <90%) limb symmetry for the single-leg, 6-m timed, triple hop, and crossover hop tests. LSI, limb symmetry index.

The median (range) LSI was 93% (41%-187%) for the single-leg hop test, 98% (52%-129%) for the 6-m timed hop test, 95% (47%-207%) for the triple-hop test, and 99% (42%-141%) for the crossover hop test. The percentage of patients with normal limb symmetry (LSI ≥90%) on the various hop tests is presented in Figure 3.

### Comparison of Strength Between Single Cruciate and Bicruciate Ligament Injuries

There were significant differences between the single and bicruciate MLKIs for quadriceps for PT/BW and TW at 60 deg/s, as well as for the 6-m timed hop test. No significant differences were found at 240 deg/s or in the other hop tests (Table 4).

**Table 4:** Outcome Scores Comparing Type 1 and Types 2-4

	Score, Mean ± SD		
Outcome Scale	Type 1	Types 2-4	*P* Value*^*a*^*
Quadriceps			
Peak torque/body weight, N·m/kg			
60 deg/s	182 ± 56	154 ± 55	**.02**
240 deg/s	110 ± 32	100 ± 39	.17
Total work, J			
60 deg/s	719 ± 250	600 ± 219	**.02**
240 deg/s	1942 ± 744	1693 ± 760	.12
Hamstring			
Peak torque/body weight, N·m/kg			
60 deg/s	87 ± 29	79 ± 31	.23
240 deg/s	56 ± 19	52 ± 22	.43
Total work, J			
60 deg/s	393 ± 153	336 ± 170	.11
240 deg/s	886 ± 377	757 ± 434	.15
Test			
Single hop, cm	94 ± 45	93 ± 45	.95
6-m timed hop, s	293 ± 125	394 ± 278	**.05**
Triple hop, cm	343 ± 123	320 ± 129	.44
Crossover hop, cm	298 ± 126	272 ± 131	.40

a Bold indicates *P*≤ .05.

Patients with single-ligament injuries demonstrated higher quadriceps LSI at 60-deg/s PT/BW (mean ± SD, 94.9 ± 18.8 vs 82.1 ± 18.2; mean difference, 12.7; *P* = .002), 60-deg/s TW (97.6 ± 22.6 vs 83.6 ± 18.2; mean difference, 14.0; *P* = .002), and 240-deg/s PT/BW (97.2 ± 13.4 vs 87.0 ± 17.4; mean difference, 10.2; *P* = .003) as compared with bicruciate ligament injuries. No significant differences were observed in hamstring LSI at any velocity (all mean differences, −0.4 to −4.1; all *P* > .36).

### Associations Among Muscle Strength, Patient-Reported Outcomes, and Return to Sport

Correlation analyses showed that quadriceps LSI at 60 deg/s was weakly but significantly correlated with International Knee Documentation Committee subjective knee form (ρ = 0.27; *P* = .011), Knee injury and Osteoarthritis Outcome Score (KOOS)–Sport and Recreation (ρ = 0.25; *P* = .022), and KOOS–Quality of Life (ρ = 0.28; *P* = .008) (Table 5). Hamstring LSI at 60 deg/s was not significantly correlated with any of the PROMs. Quadriceps LSI was not significantly associated with return to the same or higher preinjury activity level (Table 6).

**Table 5:** Correlation Between Outcome Measures and LSI for Quadriceps and Hamstrings Strength at 60 deg/s*^*a*^*

	Quadriceps LSI, 60 deg/s		Hamstrings LSI, 60 deg/s	
Outcome Measure	Spearman ρ	*P* Value	Spearman ρ	*P* Value
IKDC	0.27	.011	0.17	.121
KOOS				
Sport and recreation	0.25	.022	0.11	.300
Quality of life	0.28	.008	0.12	.282

a Spearman correlation coefficient (ρ) is reported. IKDC, International Knee Documentation Committee; KOOS, Knee injury and Osteoarthritis Outcome Score; LSI, limb symmetry index.

**Table 6:** Quadriceps LSI (60 deg/s) According to RTS Status*^*a*^*

	RTS = 1 (n = 34)	RTS = 0 (n = 53)	*P* Value
Mean ± SD	90.9 ± 23.1	86.3 ± 16.6	.281*^*b*^*
Median (IQR)	90.6 (76.9-101.6)	84.5 (73.0-98.0)	.441*^*c*^*

a LSI, limb symmetry index; RTS, return to sport.

b Independent samples *t* test.

c Mann-Whitney *U* test.

## Discussion

The most important findings from this study were that almost half of the patients undergoing MLKI surgery were not able to regain full maximal quadriceps (54%) and hamstring (46%) muscle strength as compared with the uninvolved limb at a mean 7 years postoperatively. Furthermore, 28% to 44% of patients who completed the hop tests failed to achieve a normal LSI (≥90%), suggesting that functional recovery remains incomplete for many individuals for a prolonged period after surgery. Last, patients with bicruciate injuries demonstrated significantly lower quadriceps muscle strength and inferior performance on the 6-m timed hop test as compared with patients with single cruciate injuries, indicating greater long-term impairment in more complex injury patterns.

The present study demonstrated that even at a mean 7 years after injury, a significant proportion of patients had not regained full quadriceps and hamstring muscle strength (LSI ≥90%). These findings are consistent with previous literature indicating persistent muscle deficits after an MLKI.^7,10,11,16^ Jenkins et al^16^ reported that at 2 years postoperatively, quadriceps and hamstring strength recovered to 85% and 90% of the uninjured limb, respectively. Their prospective series of 50 MLKI cases primarily included 2-ligament injuries (76%), with fewer cases involving 3 ligaments (20%) or 4 (4%). Ebert et al^10^ analyzed a cohort of 50 patients with MLKIs, of which 74% were Schenk KDI. At 24 months, the mean (SD) quadriceps LSI at 90 deg/s was 87% (10.4%), while hamstring torque LSI was better preserved at 97% (9.1%). The cohort of the present study, which was larger and followed for a longer period, supports and expands the findings of Jenkins et al and Ebert et al by showing that these strength deficits often persist well beyond 2 years postoperatively. Additionally, a systematic review^12^ of ACL reconstructions revealed that knee extensor muscle strength is meaningfully reduced (>10%) at 1 year postoperatively, with limited improvement up to and beyond 5 years after surgery, suggesting that quadriceps weakness may represent a broader challenge across different types of complex knee injuries. In the limited studies available, persistent strength deficits after MLKI reconstruction at or beyond 24 months have been reported.^7,11,22^ Gigliotakaes et al^11^ noted persistent isokinetic knee extensor deficits after a 2-stage bicruciate procedure with a mean postoperative follow-up of 27 months, while Denti et al^7^ identified isokinetic knee flexor and extensor strength deficits at 24-month follow-up in patients who underwent single-stage bicruciate reconstruction. Taken together, the findings from the present study and the available literature demonstrate that persistent strength impairments, especially for the quadriceps, are a common outcome after MLKI reconstruction.

From a clinical perspective, long-term quadriceps deficits are of particular concern, as reduced knee extensor strength has been associated with impaired function and altered joint loading. Considering the persistent muscle strength deficits observed after MLKI, it may be important to establish better long-term rehabilitation programs and critically evaluate the content of rehabilitation. Clinicians could consider incorporating exercise variation, challenging tasks, and motor learning principles to optimize rehabilitation while ensuring sufficient loading of the injured limb.^4,13,15,34^ In addition to underloading, the intensity and volume of strength training might be too low to increase muscle strength and volume to satisfactory levels. It is not clear how the use of autografts versus allografts can influence muscle recovery after surgery for complex knee injuries; however, it was beyond the scope of this study.

More than 40% of patients in the present study failed to achieve the commonly accepted LSI threshold of 90% across single-leg hop tests, indicating that functional recovery remains incomplete for a substantial proportion of individuals. In addition, approximately 16% of the patients were unable to perform the hop tests at all, whereas 3 had undergone tibialis posterior tendon transfer for their foot drop. This reflects the wide variability in recovery in this population. Research on patients after ACL reconstruction has highlighted an increased risk of reinjury among those who do not meet the commonly used 90% LSI threshold, particularly concerning quadriceps peak torque and hop performance.^14,20^ This adds clinical relevance to our findings and supports the need for rigorous, objective assessments before return to full activities. The present findings are consistent with those of Ebert et al,^10^ who also reported that many patients failed to reach the accepted 90% LSI threshold. However, the authors did not indicate the proportion of patients who reached or were unable to reach the 90% LSI threshold, making direct comparison of recovery distribution difficult. Notably, Ebert et al highlighted that hop symmetry tends to recover earlier and more fully than quadriceps strength, emphasizing a so-called strength-function mismatch. This phenomenon suggests that patients may compensate during dynamic tasks such as hopping, jumping, and running, thereby masking persistent deficits in knee extensor muscle capacity. It may also indicate that achieving full (100%) muscle strength is not required to regain normal functional properties. Biomechanical analyses have shown altered joint kinematics and compensatory movement patterns during hop tasks in patients after an ACL reconstruction, which may increase the risk of secondary injury and limit performance.^8,19^ While hop tests are valuable tools for assessing gross functional recovery, they should be interpreted alongside objective strength measurements and biomechanical analyses to characterize neuromuscular status more accurately.

The third main finding from this study is that patients with bicruciate injuries demonstrated significantly lower quadriceps strength for PT/BW and TW at 60 deg/s and inferior performance on the 6-m timed hop test as compared with patients with single cruciate injuries. This observation aligns with the growing body of evidence that injury severity strongly affects functional outcomes after an MLKI.^8,17,18,24^ Ebert et al^10^ reported similar findings with patients having KDII-KDIV injuries showing significantly lower LSI values on single- and triple-hop tests and greater quadriceps strength deficits throughout the first postoperative year. While some improvement in hop symmetry was noted by 24 months, performance remained lower than in less severe injuries.^10^ Bicruciate injuries involve simultaneous disruption of the ACL and PCL, which not only compromises knee stability to a greater extent but also increases the complexity of surgical reconstruction, often leading to more extended periods of immobilization and delayed neuromuscular recovery.^26^ Moreover, these injuries are more frequently associated with concomitant nerve injuries and postoperative complications, which may further hinder or prolong rehabilitation. Nerve injuries may cause drop foot, often necessitating orthotic devices, gait retraining, and individually adapted exercises. Such factors extend the recovery process and may contribute to delayed muscle activation, persistent strength deficits, and functional limitations. In our study, among the patients with initial nerve deficits (n = 6), 3 were unable to complete the hop tests and 4 demonstrated notable strength reduction in the involved limb; 1 patient was represented in both categories.

The H/Q ratios in this study revealed relatively preserved muscular balance in the involved limb, with median ratios ranging from 0.49 to 0.51 for PT/BW and from 0.44 to 0.51 for TW across angular velocities. These values fall within the range considered physiologically normal (typically 0.4-0.6) and suggest that, despite overall strength deficits, the relative relationship between the flexor and extensor muscle groups was maintained. These findings are supported by Gigliotakaes et al,^11^ who reported an H/Q ratio of 0.59 at 60 deg/s and 0.65 at 180 deg/s. While a balanced H/Q ratio is generally viewed as protective against reinjury, excessively high ratios may reflect disproportionate quadriceps weakness rather than true hamstring dominance.^33^ This underscores the importance of presenting not only muscle balance but also absolute strength values during follow-up assessments. In the present study, quadriceps strength demonstrated weak but significant correlations with patient-reported outcomes, including International Knee Documentation Committee form, KOOS–Sport and Recreation, and KOOS–Quality of Life, suggesting that greater limb symmetry is associated with better perceived knee function. However, no significant association was observed between quadriceps strength and return to sport. These findings indicate that while muscle strength may contribute to subjective knee function, it alone does not determine return to sport in this population. Return to sport after MLKI is likely influenced by multiple factors, including psychological readiness, injury severity, and persistent functional limitations.

The wide dispersion of strength values observed in this cohort highlights the marked heterogeneity in long-term recovery after MLKI, with some patients achieving near-complete restoration of strength, while others exhibited substantial persistent deficits. Despite statistically significant paired side-to-side differences, the variability may partly be explained by the overlapping error bars seen in Figure 1.

Some limitations of this study should be acknowledged. First, there was a substantial loss to follow-up, with nearly one-third of eligible patients being nonresponders. However, there were no significant differences between responders and nonresponders in terms of age, body mass index, injury mechanism, or injury pattern. Also, there was a large spread in age (21-72 years), indicating quite different preinjury conditions for physical function. Furthermore, the cross-sectional design limits the ability to capture individual recovery patterns or changes over time. Importantly, this study lacked detailed information on the quality, frequency, and adherence to rehabilitation, all of which are critical factors influencing the recovery of strength and function. Greater insight into its nature would be valuable for better interpreting the results and guiding clinical practice. Most injuries in this cohort were sports related, with fewer resulting from high-energy trauma such as motor vehicle accidents. As high-energy MLKIs are generally associated with more extensive tissue damage and neurovascular injury, our findings may not fully generalize to populations with a higher proportion of such injuries, where long-term strength and functional outcomes could be worse.

## Conclusion

Persistent deficits in quadriceps and hamstring muscle strength were observed 7 years after MLKI surgery, with a substantial proportion of patients failing to regain full functional symmetry in muscle strength and hop test performance. Patients with bicruciate injuries demonstrated significantly more pronounced deficits in muscle strength and hop test scores as compared with those with single cruciate injuries.

## References

[1] Andriacchi TP Briant PL Bevill SL Koo S. A framework for the in vivo pathomechanics of osteoarthritis at the knee. Ann Biomed Eng. 2004;32(3):447-457. 15095819 10.1023/b:abme.0000017541.82498.37

[2] Berg B Urhausen AP Øiestad BE , et al What tests should be used to assess functional performance in youth and young adults following anterior cruciate ligament or meniscal injury? A systematic review of measurement properties for the OPTIKNEE consensus. Br J Sports Med. 2022;56(24):1454-1464. doi:10.1136/bjsports-2022-105510 35697502

[3] Carr JB Werner BC Miller MD Gwathmey FW. Knee dislocation in the morbidly obese patient. J Knee Surg. 2016;29(4):278-286. 26838970 10.1055/s-0036-1571432

[4] Chan DK Lonsdale C Ho PY Yung PS Chan KM. Patient motivation and adherence to postsurgery rehabilitation exercise recommendations: the influence of physiotherapists’ autonomy-supportive behaviors. Arch Phys Med Rehabil. 2009;90(12):1977-1982. 19969157 10.1016/j.apmr.2009.05.024

[5] Daniel D Stone M Riehl B , et al A measurement of lower limb function: the one-leg hop for distance. Am J Knee Surg. 1988;1:212-214.

[6] Danneskiold-Samsøe B Bartels EM Bülow PM , et al Isokinetic and isometric muscle strength in a healthy population with special reference to age and gender. Acta Physiol (Oxf). 2009;197(suppl 673):1-68. 10.1111/j.1748-1716.2009.02022.x 19744082

[7] Denti M Tornese D Melegati G Schonhuber H Quaglia A Volpi P. Combined chronic anterior cruciate ligament and posterior cruciate ligament reconstruction: functional and clinical results. Knee Surg Sports Traumatol Arthrosc. 2015;23:2853-2858. doi:10.1007/s00167-015-3764-8 26318488

[8] Di Stasi SL Logerstedt D Gardinier ES Snyder-Mackler L. Muscle strength and movement patterns after ACL reconstruction. Am J Sports Med. 2013;41(6):1310-1318. 23562809 10.1177/0363546513482718 PMC3732407

[9] Dworsky-Fried J Kotipalli S Vivekanantha P , et al Rates of return to sport after surgical management of multiligament knee injuries are higher than previously described yet highly heterogeneous: a systematic review. Arthroscopy. 2025;41(6):S0749-8063(25)00429-3. doi:10.1016/j.arthro.2025.06.006 40543683

[10] Ebert JR Edwards PK Mayne AIW , et al Patients undergoing multiligament knee reconstruction injured during pivoting sports demonstrate similar clinical, functional and return to sport outcomes by 2 years compared with those undergoing anterior cruciate ligament reconstruction. Knee Surg Sports Traumatol Arthrosc. 2024;32:2967-2977. 39101299 10.1002/ksa.12409 PMC11848961

[11] Gigliotakaes I Inada MM Miranda JB Cunha SA Piedade SR. Isokinetic evaluation after two-stage bicruciate reconstruction. Acta Ortop Bras. 2014;22(1):21-24. doi:10.1590/S1413-78522014000100003 24644415 PMC3952866

[12] Girdwood M Culvenor AG Patterson B , et al No sign of weakness: a systematic review and meta-analysis of hip and calf muscle strength after anterior cruciate ligament injury. Br J Sports Med. 2024;58(9):500-510. doi:10.1136/bjsports-2023-107536 38537939

[13] Gokeler A Bisschop M Benjaminse A , et al Quadriceps function following ACL reconstruction and rehabilitation: implications for optimisation of current practices. Knee Surg Sports Traumatol Arthrosc. 2014;22(5):1163-1174. 23812438 10.1007/s00167-013-2577-x

[14] Grindem H Snyder-Mackler L Moksnes H Engebretsen L Risberg MA. Simple decision rules can reduce reinjury risk by 84% after ACL reconstruction: the Delaware-Oslo ACL cohort study. Br J Sports Med. 2016;50:804-808. doi:10.1136/bjsports-2016-096031 27162233 PMC4912389

[15] Hart JM Ko JW Konold T Pietrosimone B. Sagittal plane knee joint moments following anterior cruciate ligament injury and reconstruction: a systematic review. Clin Biomech. 2010;25(4):277-283. 10.1016/j.clinbiomech.2009.12.004 20097459

[16] Jenkins PJ Clifton R Gillespie GN Will EM Keating JF. Strength and function recovery after multiple-ligament reconstruction of the knee. Injury. 2011;42(12):1426-1432. 21481869 10.1016/j.injury.2011.03.026

[17] Kaeding CC Pedroza AD Parker RD , et al Intra-articular findings in the reconstructed multiligament-injured knee. Arthroscopy. 2005;21(4):424-430. 15800522 10.1016/j.arthro.2004.12.012

[18] Kim SH Park YB Kim BS Lee DH Pujol N. Incidence of associated lesions of multiligament knee injuries: a systematic review and meta-analysis. Orthop J Sports Med. 2021;9(6):23259671211010409. 10.1177/23259671211010409 PMC8312178 34368374

[19] Kotsifaki A Korakakis V Whiteley R Rossom S Jonkers I. Measuring only hop distance during single leg hop testing is insufficient to detect deficits in knee function after ACL reconstruction: a systematic review and meta-analysis. Br J Sports Med. 2020;54(3):139-153. doi:10.1136/bjsports-2018-099918 31142471

[20] Kyritsis P Bahr R Landreau P Miladi R Witvrouw E. Likelihood of ACL graft rupture: not meeting six clinical discharge criteria before return to sport is associated with a four times greater risk of rupture. Br J Sports Med. 2016;50:946-951. doi:10.1136/bjsports-2015-095908 27215935

[21] Lanshammar K Ribom EL. Differences in muscle strength in dominant and non-dominant leg in females aged 20-39 years: a population-based study. Phys Ther Sport. 2011;12(2):76-79. 21496769 10.1016/j.ptsp.2010.10.004

[22] LaPrade RF Chahla J DePhillipo NN , et al Single-stage multiple-ligament knee reconstructions for sports-related injuries: outcomes in 194 patients. Am J Sports Med. 2019;47(11):2563-2571. 31381372 10.1177/0363546519864539

[23] Levy BA Dajani KA Whelan DB , et al Decision making in the multiligament-injured knee: an evidence-based systematic review. Arthroscopy. 2009;25:430-438. 19341932 10.1016/j.arthro.2009.01.008

[24] Lind M Jakobsen BW Lund B Hansen MS Abdallah O Christiansen SE. Anatomical reconstruction of the medial collateral ligament and posteromedial corner of the knee in patients with chronic medial collateral ligament instability. Am J Sports Med. 2009;37(6):1116-1122. 19336612 10.1177/0363546509332498

[25] Logerstedt D Grindem H Lynch A , et al Single-legged hop tests as predictors of self-reported knee function after anterior cruciate ligament reconstruction: the Delaware-Oslo ACL cohort study. Am J Sports Med. 2012;40(10):2348-2356. 22926749 10.1177/0363546512457551 PMC3462240

[26] Moatshe G Dornan GJ Ludvigsen T , et al High prevalence of knee osteoarthritis at a minimum 10-year follow-up after knee dislocation surgery. Knee Surg Sports Traumatol Arthrosc. 2017;25(12):3914-3922. 28280907 10.1007/s00167-017-4443-8

[27] Murray IR Makaram NS Geeslin AG , et al Multiligament knee injury (MLKI): an expert consensus statement on nomenclature, diagnosis, treatment and rehabilitation. Br J Sports Med. 2024;58(23):1385-1400. doi:10.1136/bjsports-2024-108089 39237264

[28] Noyes FR Barber SD Mangine RE. Abnormal lower limb symmetry determined by functional hop tests after anterior cruciate ligament rupture. Am J Sports Med. 1991;19(5):513-518. 1962720 10.1177/036354659101900518

[29] Palmieri-Smith RM Lepley LK. Quadriceps strength asymmetry after anterior cruciate ligament reconstruction alters knee joint biomechanics and functional performance at time of return to activity. Am J Sports Med. 2015;43:1662-1669. 25883169 10.1177/0363546515578252 PMC4758854

[30] Palmieri-Smith RM Thomas AC. A neuromuscular mechanism of posttraumatic osteoarthritis associated with ACL injury. Exerc Sport Sci Rev. 2009;37(3):147-153. 19550206 10.1097/JES.0b013e3181aa6669

[31] Poploski KM Lynch AD Burns TC , et al; STaR Trial for Multiple Ligament Knee Injuries Network. Presentation and surgical management of multiple ligament knee injuries: a multicenter study from the Surgical Timing and Rehabilitation (STaR) Trial for MLKIs Network. J Bone Joint Surg Am. 2023;105(8):607-613. 36827383 10.2106/JBJS.20.02051

[32] Reid A Birmingham TB Stratford PW Alcock GK Giffin JR. Hop testing provides a reliable and valid outcome measure during rehabilitation after anterior cruciate ligament reconstruction. Phys Ther. 2007;87(3):337-349. 17311886 10.2522/ptj.20060143

[33] Rosene JM Fogarty TD Mahaffey BL. Isokinetic hamstrings:quadriceps ratios in intercollegiate athletes. J Athl Train. 2001;36(4):378-383. 12937479 PMC155432

[34] Sigward SM Chan MM Lin PE Almansouri SY Pratt KA. Compensatory strategies that reduce knee extensor demand during a bilateral squat change from 3 to 5 months following anterior cruciate ligament reconstruction. J Orthop Sports Phys Ther. 2018;48(9):713-718. 29895231 10.2519/jospt.2018.7977

[35] Taylor AR Arden GP Rainey HA. Traumatic dislocation of the knee: a report of forty-three cases with special reference to conservative treatment. J Bone Joint Surg Br. 1972;54(1):96-102. 5011750

[36] Troan I Bere T Holm I , et al Patient-reported outcomes of bicruciate multiligament versus single cruciate multiligament knee injuries. Am J Sports Med. 2025;53:138-146. 39741479 10.1177/03635465241293743 PMC11689965

[37] Troan I Bere T Holm I Loken S LaPrade RF Engebretsen L Moatshe G. Return to sport and work after multiligament knee injury. Knee Surg Sports Traumatol Arthrosc. 2026;34(1):329-337. doi:10.1002/ksa.70175 41324464

[38] Urhausen AP Berg B Øiestad BE , et al. Measurement properties for muscle strength tests following anterior cruciate ligament and/or meniscus injury: a systematic review with meta-analyses for the OPTIKNEE consensus. Br J Sports Med. 2022;56(24):1422-1431. doi:10.1136/bjsports-2022-105498 36113973

[39] Wascher DC Dvirnak PC DeCoster TA. Knee dislocation: initial assessment and implications for treatment. J Orthop Trauma. 1997;11(7):525-529. 9334955 10.1097/00005131-199710000-00011

